# Decoding the hallmarks of GLP-1RA weight-loss super-responders

**DOI:** 10.1093/biomethods/bpag021

**Published:** 2026-04-20

**Authors:** A J Venkatakrishnan, Karthik Murugadoss, Venky Soundararajan

**Affiliations:** nference, Cambridge, MA 02142, United States; Metabolism Agentic Intelligence Atlas (MAIA), Cambridge, MA 02142, United States; nference, Cambridge, MA 02142, United States; Metabolism Agentic Intelligence Atlas (MAIA), Cambridge, MA 02142, United States; nference, Cambridge, MA 02142, United States; Metabolism Agentic Intelligence Atlas (MAIA), Cambridge, MA 02142, United States

**Keywords:** GLP1 receptor agonist, weight loss, semaglutide, tirzepatide

## Abstract

Glucagon-like peptide-1 receptor agonists (GLP-1RAs) have reshaped obesity treatment, yet weight-loss outcomes remain highly uneven in real-world care. Using a federated biomedical platform, we analyzed 135 349 individuals treated with semaglutide and tirzepatide formulations and stratified them as “super responders” (>15% weight loss), “moderate responders” (5%–15% weight loss), “minimal weight-loss group” (<5% weight loss), and “weight regainers.” Ozempic, Wegovy, Mounjaro, and Zepbound had similar proportions of patients classified as moderate responders, ranging from 40% to 42%. Rates of super responders were highest for Zepbound (34%), followed by Wegovy (26%), Mounjaro (24%), and Ozempic (10%). Among moderate and super responders, the average weight after 1 year of treatment was similar to the average weight approximately 10 and 20 years prior to treatment initiation, respectively. Compared with patients with minimal or moderate response, super responders were more likely to be younger (mean age 51 years versus 55 years), female (80% versus 58%–65%), and white (90% versus 80%). Baseline clinical characteristics enriched among super responders compared to the minimal response group included fibromyalgia (rate ratio [RR]: 0.2, *P* = .002) and osteoarthritis (RR = 0.5, *P* = .001) for Zepbound, and psoriasis (RR = 2.5, *P* = .03) for Wegovy. These results highlight significant heterogeneity in weight trajectories following sustained exposure to a GLP-1RA therapy and identify factors associated with increased weight loss, likely reflecting a combination of biological, behavioral, and social factors. These insights motivate further prospective analyses to help guide the development of more tailored weight-loss intervention strategies.

## Introduction

Obesity is a rapidly escalating global public health emergency projected to affect more than 1.1 billion adults by 2030 [1], placing a growing strain on health systems worldwide. It fuels rising epidemics of diabetes, cardiovascular conditions, liver disease, and cancer, collectively responsible for more than 1.6 million premature deaths worldwide each year [1]. Despite extensive public health interventions, conventional approaches such as lifestyle modification and bariatric surgery continue to show limited and inconsistent long-term results.

The advent of glucagon-like peptide-1 receptor agonists (GLP-1RAs), originally developed for type 2 diabetes, has transformed obesity treatment [2]. From early agents such as exenatide and liraglutide to next-generation analogs including semaglutide and tirzepatide, this therapeutic class now spans multiple branded formulations with distinct dose ranges and administration regimens (Supplementary Table S1). GLP-1RAs act through central pathways that suppress appetite and enhance satiety [3], and through peripheral pathways that improve insulin secretion and glycemic control [4]. Their substantial and durable weight-loss effects, combined with cardiovascular and metabolic benefits, have positioned GLP-1RAs as a cornerstone of modern obesity care [5, 6]. Nevertheless, individual weight-loss responses vary widely [7], underscoring an urgent need for precision therapeutics. Clinical trials and real-world studies report mean weight reductions of 15%–20% in patients with obesity or type 2 diabetes mellitus [8], together with improvements in cardiovascular risk [9], yet these averages mask profound interindividual variability. Prior work has been constrained by limited longitudinal follow-up and has not defined clinically distinct responder phenotypes [9, 10]. Addressing this variability is essential to move beyond uniform treatment paradigms and to identify biological and behavioral determinants of exceptional response as well as the drivers of broader heterogeneity in weight-loss outcomes [11].

**Figure 1:**
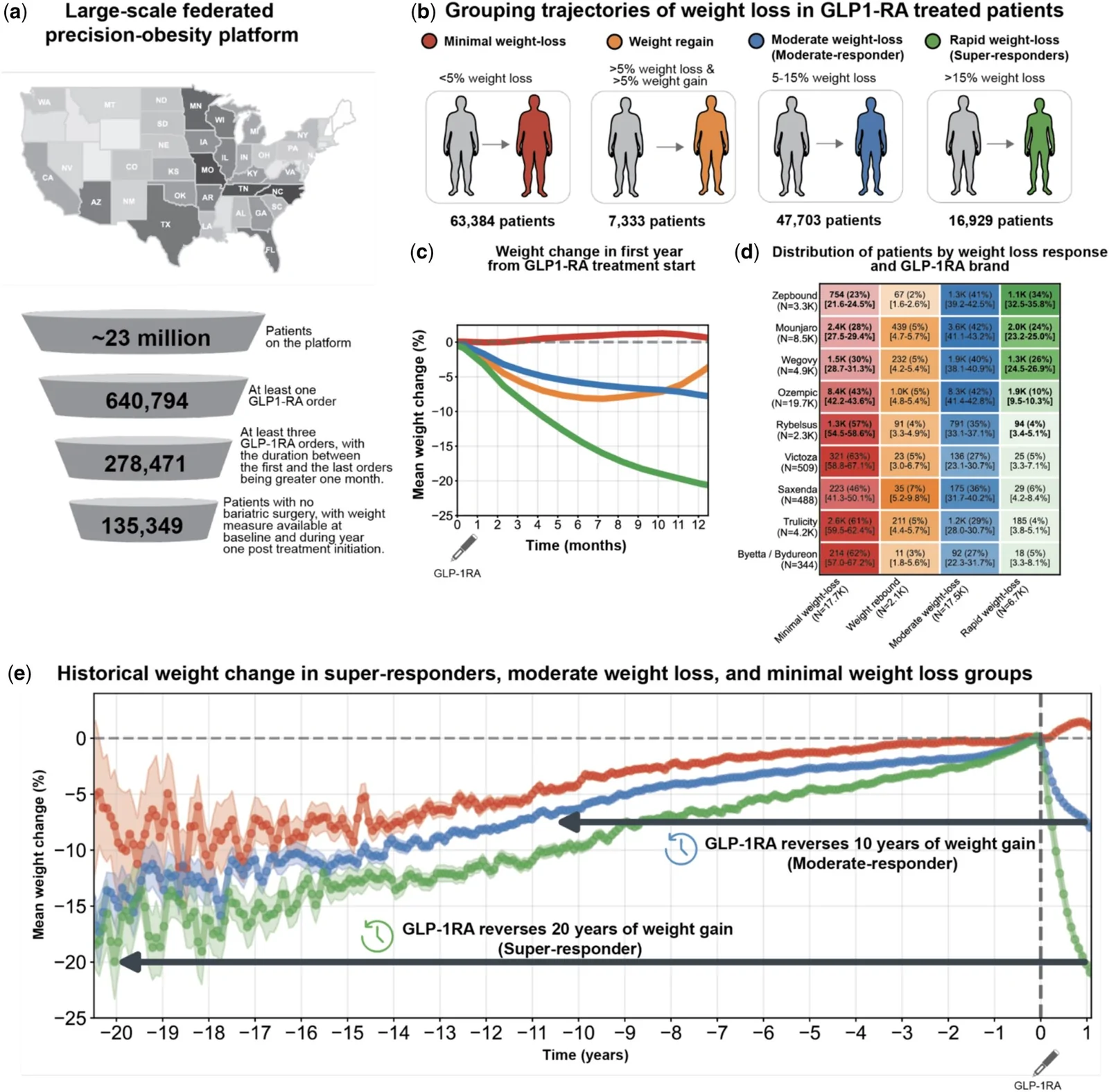
Defining GLP-1RA treatment trajectories using a large-scale precision obesity platform. (a) Overview of the federated precision obesity platform comprising approximately 23 million de-identified patient records. Of these, 640 794 patients had at least one GLP-1RA order, 278 471 met exposure criteria (≥3 prescriptions spanning ≥1 month), and 135 349 had weight measurements available before and after treatment with no history of bariatric surgery, forming the final analytic cohort. (b) Weight-loss trajectory classification in GLP-1RA-treated patients. Individuals were grouped into four categories based on weight change during the first year of GLP-1RA treatment: *minimal weight-loss group* (<5%; red), *weight regain* (>5% loss followed by >5% gain; orange), *moderate responders* (5%–15% weight loss; blue), and *super responders* (>15% weight loss, green). (c) Mean first-year post-treatment weight trajectories for the four response categories. (d) Distribution of GLP-1RA brands across the four response categories. Heatmap shows the number and percentage of patients (with 95% CI) in each category for semaglutide (Ozempic, Wegovy) and tirzepatide (Mounjaro, Zepbound) formulations, highlighting substantial brand-specific differences in response profiles. (e) Historical weight trajectories enable comparison between the magnitudes of post-treatment weight loss and pre-treatment weight gain over decades before treatment exposure shaded regions represent 95% CI.

Recent advances in electronic health records (EHRs) and AI-assisted curation of unstructured longitudinal data now enable population-level modeling of treatment trajectories [12–14]. Here, we describe a federated biomedical knowledge platform integrating 23 million de-identified US patient records to model longitudinal physiology, behavior, and therapeutic response among 135 349 individuals with a GLP-1RA prescription (Fig. 1a). To our knowledge, this represents one of the largest real-world multimodal cohorts assembled to investigate heterogeneity in GLP-1RA treatment responses. Within this cohort, we defined four distinct weight-loss trajectories during the first treatment year: super responders (>15% weight loss), moderate responders (5%–15% weight loss), minimal weight-loss group (<5% weight loss), and weight regainers. Using validated AI models for unstructured EHR curation [12, 15–18], we identified pre-treatment phenotypic signatures predictive of GLP-1RA super-response. These computable clinical phenotypes may illuminate the mechanistic basis of heterogeneity in GLP-1RA effectiveness and inform the design of next-generation precision obesity therapeutics.

## Materials and methods

### Data source and selection

This study analyzed de-identified EHR data from a network of tertiary clinical centers tied to academic medical centers in the USA through the nference nSights Analytics Platform [19, 20]. nference, in collaboration with the academic medical center (AMC) data partners, provided the de-identified data for this study. nference has established a secure data environment, hosted by and within each of the AMCs, that houses the AMC’s de-identified patient data. The provisioning of and access to this data are governed by an expert determination that satisfies the HIPAA Privacy Rule requirements for the de-identification of protected health information. Each AMC’s de-identified data environment is specifically designed and operated to enable access to and analysis of de-identified data without the need for Institutional Review Board (IRB) oversight, approval, or an exemption confirmation. Given these measures, informed consent and IRB review were not required for this study.

### Cohort eligibility criteria and study timeline

From a longitudinal database of 23 million patients, we identified 640 794 patients with at least one GLP-1RA order. To ensure adequate treatment exposure, we required patients to have at least 3 GLP-1RA orders, with the first and last orders spanning a period of at least 1 month, yielding 278 471 patients. We then excluded patients with a history of bariatric surgery (including gastric bypass, sleeve gastrectomy, gastric banding, and duodenal switch) and required the availability of weight measurements during both a baseline period (defined as no more than 90 days prior to the first GLP-1RA prescription) and during the one-year period after the first prescription. The final analytical cohort comprised 135 349 patients with weight trajectory data during the first year of GLP-1RA therapy (Fig. 1a).

### Weight trajectory analysis

Patients were classified into four response categories based on their maximum weight loss within 12 months post-treatment initiation (Fig. 1b): super responders (≥15%), moderate responders (5%–15%), minimal weight-loss group (<5%), and weight regainers (initial ≥5% loss followed by a weight regain of at least 5%). For each patient, the index date was defined as the date of the first GLP-1RA prescription, and baseline weight was determined as the measurement closest to the index date within a 90-day pre-treatment window. Percentage weight change was calculated relative to this baseline for all measurements. To generate population-level trajectories, weight measurements were aggregated into 30-day time windows (±15 days from each time point), and patient-level mean percentage changes were computed within each window (Fig. 1c and e). Patient-level mean values were calculated across patients and were then smoothed using a Savitzky–Golay filter (window length = 5, polynomial order = 3) to reduce noise while preserving temporal trends. Uncertainty was quantified using the standard error of the mean (SEM = σ/√*n*), where σ represents the standard deviation of patient-level means and *n* represents the number of patients with measurements in each time window. Shaded regions around trajectory lines represent ±1 SEM.

### GLP-1RA brand stratification analysis

To assess medication-specific effects, patients were stratified into mutually exclusive cohorts based on their GLP-1RA exposure history. “Brand-exclusive” cohorts comprised patients who received only a specific branded formulation (Ozempic, Wegovy, Rybelsus, Mounjaro, Zepbound, Victoza, Saxenda, Trulicity, Byetta, or Bydureon) without switching to any other brand or molecule. Bydureon and Bydureon BCise were combined into a single cohort given their pharmacological equivalence. The intent of this approach was to characterize weight trajectories during sustained exposure to a single therapy rather than to estimate real-world treatment effectiveness across all users. Patient distribution across response categories for each brand was visualized using heatmaps (Fig. 1d), with cell color intensity scaled independently within each response category column to highlight relative differences across medications. Each cell displayed the patient count and percentage of the drug/brand cohort, accompanied by 95% confidence intervals (95% CI) calculated using the Wilson score method for binomial proportions. Odds ratios (OR) with 95% CI were calculated to compare treatment response categories between GLP-1 receptor agonist formulations. Statistical significance was assessed using the Wald test for log-transformed odds ratios. *P*-values < .05 were considered statistically significant.

### Propensity score matching

To mitigate demographic imbalance between super responders, moderate responders, and the minimal weight-loss group, propensity score matching was performed using age and gender as matching variables. Of note, this matching was not intended to be a comprehensive adjustment for confounding variables but rather to reduce large demographic differences between the cohorts. Propensity scores were estimated using logistic regression with a maximum of 1000 iterations and the Limited-memory Broyden-Fletcher-Goldfarb-Shanno (L-BFGS) optimization algorithm, with treatment status (super responders versus minimal weight-loss group or super responders versus moderate responders) as the outcome. Gender was encoded as a binary variable (male = 1, female = 0), and patients with missing values in either matching variable were excluded prior to score estimation. Greedy nearest-neighbor matching without replacement was conducted with a 1:1 matching ratio and a caliper width of 0.1 (maximum absolute difference in propensity scores between matched pairs). For each super responder, the nearest available patient from the minimal weight-loss (or moderate weight-loss) cohort within the caliper was selected, and that patient was removed from the matching pool. Super responders who could not be matched within the caliper were excluded from subsequent trajectory analyses. This approach yielded balanced cohorts with minimized baseline age and gender differences between weight loss groups.

### Summary statistics and statistical testing

Summary statistics were generated for the overall cohort as well as for drug-exclusive and brand-exclusive cohorts, with analyses performed separately for propensity-matched and unmatched populations (Supplementary Tables S2–S7). Demographic distributions, including age (at first GLP-1RA prescription), gender, and race (White, Black/African American, Hispanic, Other/Unknown), were assessed across all cohorts. Baseline clinical characteristics included pregnancy status, Type 2 diabetes mellitus (T2DM) status, body mass index (BMI), and weight, defined as measurements obtained within 90 days prior to the first prescription. Pregnancy status was defined as the presence of one or more pregnancy-associated diagnoses documented in the 1-year period preceding the first GLP-1 RA prescription. T2DM status was defined as the presence of three or more diabetes diagnoses documented in the 5-year period preceding the first GLP-1 RA prescription. The distribution of weight measurements per patient was characterized across both the pretreatment and 1-year post-treatment period. The time interval in months between the first and last GLP-1RA prescription was calculated for each patient, and prescription frequency was quantified as the total number of prescriptions received during follow-up. To assess data completeness, baseline monthly clinical notes per patient (quantified over the year prior to the index date) and post-treatment monthly clinical notes per patient (quantified over the 12 months following the index date) were calculated. Categorical comparisons between response groups, including gender distribution, T2DM status, pregnant patient status, and race/ethnicity, were evaluated using counts (*N*) and percentages (%), with chi-square tests used for equality of proportions. Statistical significance for categorical comparisons was assessed using *P*-values, with *P* < .05 considered statistically significant. For continuous variables, means with standard deviations (SD) and medians with interquartile ranges (IQR 25th and 75th percentiles) were calculated.

### Augmented curation

We employed a multi-stage information extraction (IE) pipeline to extract clinical phenotypes, such as patient diseases and comorbidities, from unstructured clinical notes. The pipeline’s architecture combined fine-tuned Bidirectional Encoder Representations from Transformers (BERT) [21] models. The workflow first employs a Named Entity Recognition (NER) model to identify clinical entities, including diseases, symptoms, and medications. Subsequently, a series of sequential qualifier models assesses the clinical context for each extracted entity by determining its subject (patient versus other), temporality (current, past, or hypothetical), and certainty (confirmed, negated, or suspected). This contextualization framework is essential for disambiguating confirmed, current patient symptoms (e.g. “Patient reports persistent nausea”) from negated findings (e.g. “No vomiting”), suspected conditions (e.g. “Rule out gastroparesis”), or prior diagnoses (e.g. “Had sleep apnea last year”). This IE approach has been validated in prior studies [15], demonstrating high fidelity in capturing conditions and medications from clinical notes with F1 scores of 0.93 and 0.95, respectively.

### Comparative analysis of phenotypes in weight loss cohorts

We performed a comparative analysis of disease prevalence by assessing patient cohorts at different time points and against each other across a total set of 1426 unique diseases. The analysis utilized both structured diagnoses and unstructured phenotypes. These phenotypes were extracted from all available clinical documentation within the defined pre-treatment (1 year to 30 days prior to index) period, restricting the output to confirmed, current patient-attributed diseases. To identify potential predictive factors for GLP-1RA responsiveness, pre-treatment disease prevalence was compared between super responders and the minimal weight loss or moderate response group. These comparisons were performed independently for four GLP-1RA medications (Zepbound, Mounjaro, Wegovy, and Ozempic). The prevalence of each disease phenotype was compared by calculating a Rate Ratio (RR) and a chi-square test *P*-value, corrected for False Discovery Rate (FDR) using the Benjamini-Hochberg (BH) procedure. For heatmap visualization, a subset of features was selected by identifying diseases that showed both statistically significant associations (FDR-corrected *P* < .05) and demonstrated strong effect sizes (RR > 2.0 or RR < 0.5) in at least one GLP-1RA medication. To construct the final disease list for each heatmap, a maximum of five diseases with the highest RRs and five diseases with the lowest RRs were selected per drug.

### De-identification and HIPAA compliance certification

Prior to analysis, all EHR data were de-identified under an expert determination consistent with the Health Insurance Portability and Accountability Act (HIPAA) Privacy Rule [45 CFR §164.514(b)(1)]. The de-identification methodology employed a multi-layered transformation approach to both structured and unstructured data fields [19, 20]. In structured data, direct identifiers including patient names and precise geographic locations were excluded entirely, while indirect identifiers underwent specific transformations: patient identifiers, medical record numbers, and accession numbers were replaced with one-way cryptographic hashes using confidential salts to preserve linkage across patient encounters; all dates were shifted backward by patient-specific random offsets (1–31 days) to preserve temporal relationships while obscuring exact event timing; the ZIP codes were truncated to two-digit state-level resolution; and continuous variables including age, height, weight, and body mass index were thresholded to prevent identification of extreme values (for example, ages ≥89 years transformed to “89+” and BMI >40 transformed to “40+”). In unstructured clinical text, an ensemble de-identification system that combines attention-based deep learning models with rule-based methods achieved an estimated >99% recall for personally identifiable information (PII) detection, with detected identifiers replaced by plausible fictional surrogates [19].

### Data harmonization

To address heterogeneity in EHR data, we harmonized clinical variables, including medications, anthropometric measurements, and diagnoses, to standardized concepts. For medications, we first constructed a standardized drug concept database combining the nSights knowledge graph with RXNorm (https://www.nlm.nih.gov/research/umls/rxnorm/index.html) hierarchies to capture ingredient, brand, and dose-specific information. EHR medication records were matched using a hierarchical approach, prioritizing RXNorm codes when available, followed by ingredient-level matching, and finally natural language processing and pattern matching on free-text medication orders when structured codes were absent. For anthropometric measurements (height, weight, BMI), we created a unified vocabulary from SNOMED (https://www.snomed.org/, https://athena.ohdsi.org) and LOINC (https://loinc.org/) terminologies and matched EHR measurement descriptions using standardized text matching algorithms with abbreviation expansion and synonym resolution; ambiguous mappings were resolved using OpenAI GPT-4o (https://platform.openai.com/docs/models/gpt-4o) with summary statistics as context, followed by manual verification. For diagnoses, we developed a hierarchical disease concept database from the nSights knowledge graph and matched EHR diagnosis descriptions and codes by identifying the most specific common child concept in the hierarchy. This approach enabled consistent identification of clinical entities while preserving granularity where available.

## Results

### Characterization of heterogeneity in weight change trajectories after GLP-1RA initiation

We stratified 135 349 GLP-1RA-treated patients into four weight-loss response categories (Fig. 1b): minimal weight-loss (<5%, *N* = 63 384), weight regain (>5%gain, *N* = 7333), moderate weight-loss (5%–15%, *N* = 47 703), and rapid weight-loss or “super responders” (>15%, *N* = 16 929). Mean weight trajectories diverged within the first three months of treatment, after which the minimal weight-loss group remained weight-stable, moderate responders reached a shallow plateau, super responders continued to lose weight throughout the year, and the weight regain group showed a reversal of early weight loss (Fig. 1c). Super responders achieved an average reduction of approximately 18% of baseline body weight by 1 year, compared with roughly 10% in moderate responders and negligible change in the minimal weight-loss group. These trajectories highlight pronounced interindividual variability in real-world GLP-1RA effectiveness and reveal distinct physiological response patterns that emerge early in treatment.

Response distributions differed markedly across GLP-1RA formulations and branded products (Fig. 1d; Supplementary Table S2). Zepbound (1118 super responders, 754 minimal weight-loss group) showed significantly higher odds of super-response (OR = 1.47, 95% CI 1.33–1.61, *P* < .001) and lower odds of minimal weight-loss (OR = 0.70, 95% CI 0.63–0.77, *P* < .001) relative to Wegovy (1258 super responders, 1468 minimal weight-loss group). Mounjaro (2039 super responders, 2410 minimal weight-loss group) showed markedly higher odds of super-response (OR = 2.84, 95% CI 2.65–3.04, *P* < .001) and lower odds of minimal weight-loss (OR = 0.52, 95% CI 0.49–0.54, *P* < .001) compared with Ozempic (1941 super responders, 8436 minimal weight-loss group). Moderate-response rates remained consistent across brands, ranging from 40% to 42% (41% for Zepbound, 42% for Mounjaro, 40% for Wegovy, and 42% for Ozempic), indicating that moderate weight loss was the predominant response phenotype. Earlier GLP-1RAs such as liraglutide (Saxenda) and dulaglutide (Trulicity) were dominated by the minimal weight-loss group in the real-world setting (Fig. 1d).

### Baseline demographic and clinical associations with GLP-1RA response categories

Compared to moderate responders and the minimal weight-loss group, super responders were modestly younger (mean age approximately 51 years versus 55 years, respectively) and predominantly female (approximately 80% versus 58%–65%, respectively), with the highest female representation observed in Zepbound (82.5%) and Wegovy (88.3%) cohorts (Supplementary Table S2) in super responders. Across response groups, approximately 2% of patients were pregnant in the year prior to GLP-1RA initiation, a factor that may influence baseline weight trajectories. Racial and ethnic distributions differed across response categories, with Caucasian patients enriched among super responders compared to minimal weight-loss patients (90% versus 80%, respectively), whereas African American (2.2% versus 9.1%) and Hispanic patients (1.1% versus 5.1%) were correspondingly depleted among super responders compared with minimal weight-loss patients (Supplementary Table S2). Baseline body mass index (BMI) was similar across response groups at approximately 36 kg/m^2^. Type 2 diabetes prevalence was similar between super responders and the minimal weight-loss group for Mounjaro (39.8% versus 39.9%) and Ozempic (49.3% versus 52.0%), whereas Zepbound (<1%) and Wegovy (<2%) cohorts showed substantially lower diabetes prevalence across all response groups, consistent with their predominant use in obesity without diabetes (Supplementary Table S3). Together, these demographic and clinical patterns reveal distinct response-enriched patient segments across GLP-1RA brands and underscore the opportunity for more individualized therapeutic selection.

Differences in treatment outcomes could partly reflect variability in treatment exposure, including treatment compliance, duration of prescription, and prescription frequency (Supplementary Fig. S1, Supplementary Table S4). Ozempic (approved December 2017) users had median prescription durations of approximately 14–15 months with 8–9 prescriptions per patient across response categories. Wegovy (approved June 2021) showed median prescription durations of roughly 8–12 months with 6–11 prescriptions per patient. Tirzepatide-based therapies Mounjaro (approved May 2022) and Zepbound (approved November 2023) had shorter median prescription durations of about 6–12 months, with 6–14 prescriptions per patient. Clinical engagement, measured by monthly documentation frequency, remained similar across GLP-1RAs and response groups before and after treatment, at a median of 2–4 clinical documents per patient per month (Supplementary Table S4).

### Differences in pre-treatment weight trajectories among GLP-1RA response categories

Longitudinal reconstruction of pre-treatment body-weight trajectories revealed that some patients taking GLP-1RA therapy experienced weight loss that restored prior weights dating back over a decade (Fig. 1e). For example, the average post-treatment weight of super responders was similar to the average weight approximately 20 years before therapy initiation, and the average post-treatment weight of moderate responders was similar to the average weight 10 years before initiation. In contrast, the minimal weight-loss group showed nearly flat pre-treatment trajectories. A modest late-year weight decline was observed, warranting continued monitoring of the minimal weight-loss group into the second treatment year, particularly for Mounjaro, Zepbound, and Wegovy (Supplementary Figs S2 and S3).

### Pre-treatment disease signatures reveal brand-specific physiological entry points to GLP-1RA response trajectories

**Figure 2:**
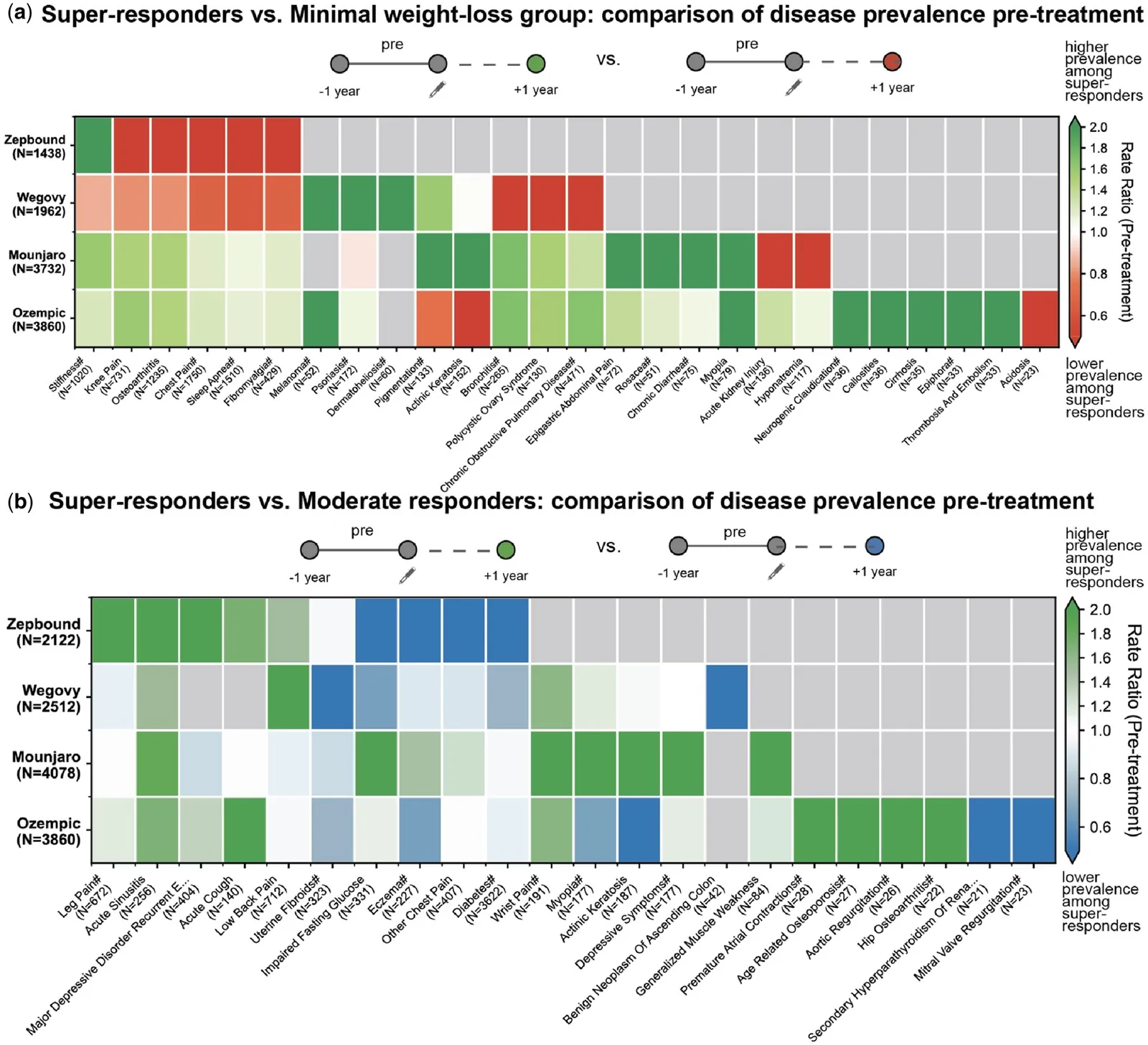
Pre-treatment disease signatures distinguishing GLP-1RA super responders from moderate and minimal weight-loss groups. (a) Heatmap comparing pre-treatment disease prevalence between super responders and the minimal weight-loss group across four GLP-1RA brands (Zepbound, Wegovy, Mounjaro, Ozempic). Each cell displays the pre-treatment rate ratio (RR), calculated as the underlying prevalence in super responders divided by the underlying prevalence in the minimal weight-loss group in the 1 year prior to GLP-1RA treatment initiation. Green shading indicates RR > 1 and red shading indicates RR < 1. Row *N* values represent total cohort size for each propensity-matched comparison (matched pairs × 2), and column *N* values represent the total pre-treatment prevalence count for each disease across all four brands. Diseases labeled with “#” denote text-derived diagnoses identified from AI-curated clinical notes. (b) Heatmap comparing pre-treatment disease prevalence between super responders and the moderate responders across four GLP-1RA brands. Each cell displays the pre-treatment rate ratio (RR), calculated as the underlying prevalence in super responders divided by the underlying prevalence in the moderate responders in the 1 year prior to GLP-1RA treatment initiation. Green shading indicates RR >1 and blue shading indicates RR < 1. Row *N* values represent total cohort size for each propensity-matched comparison (matched pairs × 2), and column *N* values represent the total pre-treatment prevalence count for each disease across all four brands. Diseases labeled with “#” denote text-derived diagnoses identified from AI-curated clinical notes.

To investigate whether differential pre-treatment health profiles predispose patients to divergent GLP-1RA outcomes, we performed propensity-matched comparisons of pre-treatment disease prevalence between: (1) super responders versus moderate responders and (2) super responders versus minimal weight-loss patients. The cohort pairs were propensity-matched exclusively on age and sex to mitigate significant demographic imbalances between the cohorts (see “Materials and Methods” section). Cohort pairs demonstrated comparable baseline characteristics, including pregnancy rates (Supplementary Table S5), BMI (Supplementary Table S6), type 2 diabetes prevalence (Supplementary Table S6), and rates of clinical engagement (Supplementary Table S7). Brand-specific pre-treatment signatures emerged that differentiated super responders from both minimal and moderate responders (Fig. 2a and b).

Compared with the Zepbound minimal weight-loss cohort (*N* = 719 matched pairs), Zepbound super responders had higher pre-treatment prevalence of muscle stiffness (RR = 2.4, *P* = .037) and lower prevalence of fibromyalgia (RR = 0.2, *P* = .002), knee pain (RR = 0.5, *P* = .014), chest pain (RR = 0.4, *P* < .001), osteoarthritis (RR = 0.5, *P* = .001), and obstructive sleep apnea (RR = 0.4, *P* < .001; Fig. 2a). For the Wegovy cohorts, (*N* = 981 matched pairs), super responders had significantly higher pre-treatment prevalence for psoriasis (RR = 2.5, *P* = .033), dermatoheliosis (RR = 2.0, *P* = .044), and melanoma (RR = 4.4, *P* = .008; Fig. 2a), and lower pre-treatment prevalence for bronchitis (RR = 0.2, *P* < .001), chronic obstructive pulmonary disease (RR = 0.2, *P* < .001), and polycystic ovarian syndrome (RR = 0.2, *P* < .001).

Among Mounjaro patients (*N* = 1866 matched pairs), super responders had higher pre-treatment prevalence of myopia (RR = 2.9, *P* = .01), chronic diarrhea (RR = 5.4, *P* = .001), epigastric abdominal pain (RR = 5.4, *P* = .001), rosacea (RR = 5.4, *P* = .001), and actinic keratosis (RR = 7.4, *P* < .001), and lower prevalence of acute kidney injury (RR = 0.34, *P* = .010) and hyponatremia (RR = 0.37, *P* = .029; Fig. 2a). Finally, in the Ozempic cohorts (*N* = 1930 matched pairs), super responders had significantly higher pre-treatment prevalence of myopia (RR = 7.2, *P* < .001), neurogenic claudication (RR = 7.2, *P* < .001), callosities (RR = 7.2, *P* < .001), cirrhosis (RR = 7.0, *P* < .001), epiphora (RR = 6.6, *P* < .001), and embolism (RR = 6.6, *P* < .001), and lower pre-treatment prevalence of acidosis (RR = 0.2, *P* = .006) and actinic keratosis (RR = 0.3, *P* = .002; Fig. 2a).

The analyses comparing super responders versus moderate responders for each medication are presented in Fig. 2b.

## Discussion

In the new era of incretin-based obesity pharmacotherapy, understanding and leveraging the marked heterogeneity in individual responses is critical for optimizing therapeutic selection. This study demonstrates the striking interindividual variability in response to GLP-1RAs, with outcomes ranging from weight regain and minimal weight loss to super responders who experience more than 15% weight loss on the same agents. Considering this high degree of response variability, this study suggests that precision stratification, rather than expanded access alone, will be important to optimize the impact of incretin therapies at the population level.

To move toward precision stratification, it is important to understand features that are associated with responsiveness to GLP-1RAs. To our knowledge, this is the largest study to explore the association between baseline clinical and demographic characteristics and weight loss after GLP-1RA initiation. In this study, super responders were more likely to be younger, female, and white. Importantly, these findings do not imply that these features must be biological drivers of treatment response but rather capture real-world associations that could be related to multiple underlying and interacting factors, including but not limited to the likelihood of treatment adherence and social determinants of health. Our analyses of pre-treatment disease diagnoses also highlighted phenotypes associated with the degree of weight loss. For example, Zepbound super responders showed lower baseline sleep apnea prevalence compared to patients who achieved minimal or moderate weight loss. Interestingly, in the phase 3 SURMOUNT-OSA trial [22], tirzepatide was shown to reduce the apnea–hypopnea index in individuals with obesity and obstructive sleep apnea. Our findings and the clinical trial results motivate further research into the relationship between GLP-1 biology and sleep apnea.

Strikingly, the average post-treatment weight in super responders matched the average weight from 20 years prior to GLP-1RA exposure, illustrating the profound benefit achieved by some patients. Recent literature suggests that GLP-1RAs may modulate aging-related physiology across multiple organ systems [23]. Interestingly, a recent body-wide multi-omic analysis of male mice treated with exenatide demonstrated a counteraction of “molecular age” which was dependent on hypothalamic expression of GLP-1RA [24]. Independent of GLP-1RA therapies, other efforts have enabled the derivation of tissue-agnostic and tissue-specific aging signatures from transcriptomic data [25] along with functional “organ ages” from multimodal data types, including cardiac age from electrocardiograms, brain age from magnetic resonance (MR) images of the brain, and abdominal age from MR images of the liver and pancreas [26]. Our findings also motivate further inquiry into the existence and characterization of “metabolic age” and the mechanisms by which GLP-1RAs may impact these various markers of non-chronologic age [27].

This study has limitations. First, it is a retrospective analysis of real-world EHR data, which is subject to various biases and data incompleteness. Restriction of the analysis to patients with multiple prescriptions for a single GLP-1RA agent introduces survivor bias and may overestimate tolerability and effectiveness compared with broader real-world populations that include patients who discontinue the medication or switch to another agent. The intent of this design was to characterize weight trajectories during sustained exposure to a single therapy rather than to estimate “intent-to-treat” effectiveness across all users in the real-world setting. Second, the study design did not account for differences in dosing, titration patterns, or medication adherence, which influence real-world effectiveness (Supplementary Fig. S4) [28, 29]. Third, the study did not specifically assess or account for the impact of most clinical comorbidities, concurrent therapies, or socioeconomic status on GLP-1RA responsiveness. While BMI distributions and rates of type 2 diabetes diagnosis were similar between response groups for single medications, this study was not designed as a comparative effectiveness study, so direct comparison of weight change trajectories between medications will inherently reflect both differences in pharmacologic effectiveness and the underlying characteristics of the treated patient populations. For example, the higher rates of type 2 diabetes in patients treated with Mounjaro or Ozempic compared to Zepbound or Wegovy are expected given the different indications and typical clinical use cases for these medications. Fourth, the study did not account for diet quality, levels of physical activity, or sleep patterns, all of which can play an important role in weight modulation, including in response to GLP-1Ras [30–32]. While the seminal clinical trials evaluating semaglutide and tirzepatide for weight loss have uniformly incorporated physical activity as an intervention [5], the prescription of physical activity and adherence to such recommendations in the real-world setting are likely to be far more variable, which could certainly contribute to the heterogeneous weight trajectories observed in our study. Some information regarding these variables can be garnered from unstructured EHR content (i.e. clinical notes and questionnaires), but this study motivates more focused analyses that routinely collect such data (e.g. from wearable devices) and evaluate their association with on-treatment weight trajectories. Finally, the comparison of pre-treatment diseases between the response groups used a combination of structured disease codes and natural language processing to determine disease prevalence. There are limitations to both the sensitivity and specificity of structured codes in assessing disease prevalence, and we and others have shown that consideration of unstructured data (i.e. curation of clinical notes) can significantly improve these metrics [33, 34]. That said, clinical notes have shortcomings as well, including the fact that documentation bias likely impacts the estimate of true clinical incidence.

In conclusion, this large-scale assessment of weight trajectories in patients with sustained exposure to a GLP-1RA therapy highlights significant heterogeneity in individual-level responses and identifies baseline features that are enriched in patients with significant weight loss compared to those with minimal weight loss. This study motivates further exploration of various axes that modulate GLP-1RA effectiveness, including biological and behavioral mechanisms that can be exploited to design more effective treatment selection and maintenance strategies.

## Supplementary Material

bpag021_Supplementary_Data

## Data Availability

This study involves the analysis of de-identified EHR data via the nference nSights Federated Clinical Analytics Platform (nSights). Data shown and reported in this manuscript were extracted from this environment using an established protocol for data extraction, aimed at preserving patient privacy. The data has been de-identified pursuant to an expert determination in accordance with the HIPAA Privacy Rule. Any data beyond what is reported in the manuscript, including but not limited to the raw EHR data, cannot be shared or released due to the parameters of the expert determination to maintain the data de-identification. Contact the corresponding author for additional details regarding nSights.
