# Supplementary material for: Decoding the hallmarks of GLP-1RA weight-loss super-responders
Source: Biol Methods Protoc. 2026 Apr 20;11(1):bpag021. doi: 10.1093/biomethods/bpag021 (PMC13176449; doi:10.1093/biomethods/bpag021)
Supplement: bpag021_Supplementary_Data [file bpag021_supplementary_data.docx]

## **Supplementary Information**

**Table S1**. Brand details for the GLP-1RAs included in this study.

| **Brand** | **Drug** | **Approval Date** | **Min Dose (mg)** | **Max Dose (mg)** | **Route** | **Frequency** |
| --- | --- | --- | --- | --- | --- | --- |
| Ozempic | Semaglutide | Dec 2017 | 0.25 | 2 | Subcutaneous | Weekly |
| Wegovy | Semaglutide | June 2021 | 0.25 | 2.4 | Subcutaneous | Weekly |
| Mounjaro | Tirzepatide | May 2022 | 2.5 | 15 | Subcutaneous | Weekly |
| Zepbound | Tirzepatide | Nov 2023 | 2.5 | 15 | Subcutaneous | Weekly |
| Saxenda | Liraglutide | Dec 2014 | 0.6 | 3 | Subcutaneous | Daily |
| Victoza | Liraglutide | Jan 2010 | 0.6 | 1.8 | Subcutaneous | Daily |
| Trulicity | Dulaglutide | Sept 2014 | 0.75 | 4.5 | Subcutaneous | Weekly |
| Byetta | Exenatide | Apr 2005 | 5 | 20 | Subcutaneous | Twice Daily |
| Bydureon | Exenatide | Jan 2012 | 0 | 2 | Subcutaneous | Weekly |
| Rybelsus | Semaglutide | Sept 2019 | 3 | 14 | Oral | Daily |

**Table S2.** Demographic characteristics of non–propensity-matched cohorts of weight-loss super responders, moderate responders, the minimal weight-loss group, and the weight regain group across GLP-1RA drugs and brands.

| **DRUG**  **(Active Compound)** | **BRAND (Marketed Formulation)** | **Weight-Loss Outcome Category** | **Number of Patients (N)** | **Male (N)** | **Female (N)** | **Male Percentage (%)** | **Gender Association –log₁₀(P)** | **Age in years,**  **Mean (SD)** | **Age in years,**  **Median [IQR]** | **Pregnant Patients N (%)** | **Race: White N (%)** | **Race: Black/African American N (%)** | **Race: Hispanic N (%)** | **Race: Other/Unknown N (%)** |
| --- | --- | --- | --- | --- | --- | --- | --- | --- | --- | --- | --- | --- | --- | --- |
| **All GLP-1 Receptor Agonists** |  | Minimal weight-loss group | 63753 | 26207 | 37538 | 41.1 | inf | 54.58 (13.50) | 56 [45, 65] | 678 (1.1%) | 52582 (83.0%) | 5799 (9.1%) | 1366 (2.2%) | 3637 (5.7%) |
| **All GLP-1 Receptor Agonists** |  | Weight regain | 7365 | 2497 | 4867 | 33.9 | 167.17 | 52.09 (14.08) | 52 [42, 63] | 139 (1.9%) | 6362 (86.8%) | 521 (7.1%) | 119 (1.6%) | 331 (4.5%) |
| **All GLP-1 Receptor Agonists** |  | Moderate Responder | 47196 | 16817 | 30376 | 35.6 | inf | 55.28 (13.60) | 57 [46, 66] | 479 (1%) | 40447 (85.9%) | 3502 (7.4%) | 867 (1.8%) | 2257 (4.8%) |
| **All GLP-1 Receptor Agonists** |  | Super Responder | 16950 | 3375 | 13573 | 19.9 | inf | 51.52 (13.58) | 52 [41, 62] | 229 (1.4%) | 15247 (90.1%) | 867 (5.1%) | 178 (1.1%) | 637 (3.8%) |
| **Semaglutide** | (Ozempic / Wegovy / Rybelsus) | Minimal weight-loss group | 16122 | 7284 | 8836 | 45.2 | 33.63 | 56.15 (13.87) | 57 [47, 66] | 106 (1.7%) | 8190 (82.7%) | 756 (7.6%) | 369 (3.7%) | 589 (5.9%) |
| **Semaglutide** | (Ozempic / Wegovy / Rybelsus) | Weight regain | 1960 | 751 | 1208 | 38.3 | 24.27 | 53.86 (14.62) | 55 [43, 66] | 31 (1.6%) | 1072 (87.2%) | 73 (5.9%) | 16 (1.3%) | 68 (5.5%) |
| **Semaglutide** | (Ozempic / Wegovy / Rybelsus) | Moderate Responder | 14819 | 5731 | 9087 | 38.7 | 166.59 | 57.63 (13.73) | 59 [49, 68] | 77 (0.5%) | 8866 (86.8%) | 622 (6.1%) | 235 (2.3%) | 491 (4.8%) |
| **Semaglutide** | (Ozempic / Wegovy / Rybelsus) | Super Responder | 4601 | 889 | 3710 | 19.3 | inf | 53.47 (14.48) | 54 [43, 65] | 47 (1.0%) | 2907 (91.2%) | 125 (3.9%) | 27 (0.8%) | 129 (4.0%) |
| **Tirzepatide** | (Mounjaro / Zepbound) | Minimal weight-loss group | 4474 | 1684 | 2789 | 37.6 | 60.59 | 52.42 (13.69) | 53 [43, 63] | 55 (1.2%) | 2544 (80.4%) | 319 (10.1%) | 98 (3.1%) | 203 (6.4%) |
| **Tirzepatide** | (Mounjaro / Zepbound) | Weight regain | 794 | 210 | 584 | 26.4 | 39.48 | 48.81 (13.42) | 49 [39, 59] | 31 (3.9%) | 450 (86.7%) | 18 (3.5%) | 17 (3.3%) | 34 (6.6%) |
| **Tirzepatide** | (Mounjaro / Zepbound) | Moderate Responder | 7345 | 2731 | 4613 | 37.2 | 106.17 | 53.47 (13.43) | 54 [44, 64] | 61 (0.8%) | 4141 (84.5%) | 390 (8.0%) | 125 (2.5%) | 247 (5.0%) |
| **Tirzepatide** | (Mounjaro / Zepbound) | Super Responder | 5367 | 1164 | 4203 | 21.7 | inf | 50.65 (13.03) | 50 [41, 60] | 49 (0.9%) | 2811 (89.0%) | 155 (4.9%) | 54 (1.7%) | 137 (4.3%) |
| (Semaglutide) | **Ozempic** | Minimal weight-loss group | 8436 | 4405 | 4031 | 52.2 | 4.33 | 58.14 (12.98) | 60 [50, 67] | 50 (0.6%) | 6960 (82.5%) | 627 (7.4%) | 347 (4.1%) | 502 (5.9%) |
| (Semaglutide) | **Ozempic** | Weight regain | 1006 | 442 | 563 | 44 | 3.87 | 57.22 (13.35) | 59 [48, 67] | <11 (<1%) | 870 (87.3%) | 63 (6.3%) | 16 (1.6%) | 48 (4.8%) |
| (Semaglutide) | **Ozempic** | Moderate Responder | 8278 | 3586 | 4692 | 43.3 | 33.27 | 60.16 (12.54) | 62 [53, 69] | 31 (0.4%) | 7154 (86.4%) | 499 (6.0%) | 235 (2.8%) | 390 (4.7%) |
| (Semaglutide) | **Ozempic** | Super Responder | 1941 | 495 | 1446 | 25.5 | 102.61 | 59.11 (13.01) | 61 [50, 68] | <11 (<1%) | 1748 (90.6%) | 84 (4.4%) | 27 (1.4%) | 71 (3.7%) |
| (Tirzepatide) | **Mounjaro** | Minimal weight-loss group | 2410 | 1061 | 1348 | 44 | 8.3 | 55.06 (13.37) | 56 [46, 65] | 20 (0.8%) | 1230 (83.8%) | 129 (8.8%) | 22 (1.5%) | 87 (5.9%) |
| (Tirzepatide) | **Mounjaro** | Weight regain | 452 | 139 | 313 | 30.8 | 15.56 | 50.67 (13.46) | 51 [41, 60] | 10 (2.2%) | 202 (87.1%) | 10 (4.3%) | <11 (<1%) | 20 (8.6%) |
| (Tirzepatide) | **Mounjaro** | Moderate Responder | 3565 | 1535 | 2030 | 43.1 | 15.95 | 56.42 (12.83) | 57 [48, 66] | 20 (0.6%) | 1712 (88.4%) | 123 (6.4%) | <11 (<1%) | 101 (5.2%) |
| (Tirzepatide) | **Mounjaro** | Super Responder | 2039 | 524 | 1515 | 25.7 | 106.03 | 53.93 (12.94) | 55 [45, 64] | 16 (0.8%) | 1159 (92.1%) | 41 (3.3%) | <11 (<1%) | 58 (4.6%) |
| (Semaglutide) | **Wegovy** | Minimal weight-loss group | 1468 | 391 | 1076 | 26.7 | 70.81 | 48.16 (14.37) | 48 [38, 58] | 15 (1%) | 1928 (80.0%) | 249 (10.3%) | 83 (3.4%) | 150 (6.2%) |
| (Semaglutide) | **Wegovy** | Weight regain | 239 | 60 | 179 | 25.1 | 13.86 | 46.62 (14.04) | 45 [36, 58] | 10 (4.3%) | 388 (85.8%) | 13 (2.9%) | 17 (3.8%) | 34 (7.5%) |
| (Semaglutide) | **Wegovy** | Moderate Responder | 1936 | 546 | 1389 | 28.2 | 81.13 | 49.81 (14.33) | 50 [40, 60] | 22 (1.1%) | 3009 (84.4%) | 273 (7.7%) | 102 (2.9%) | 181 (5.1%) |
| (Semaglutide) | **Wegovy** | Super Responder | 1258 | 179 | 1077 | 14.3 | 140.92 | 46.93 (13.53) | 47 [37, 56] | 23 (1.8%) | 1814 (89.0%) | 107 (5.2%) | 37 (1.8%) | 81 (4.0%) |
| (Tirzepatide) | **Zepbound** | Minimal weight-loss group | 754 | 209 | 545 | 27.7 | 33.7 | 47.82 (13.67) | 48 [37, 58] | 19 (2.5%) | 616 (81.7%) | 70 (9.3%) | 15 (2.0%) | 53 (7.0%) |
| (Tirzepatide) | **Zepbound** | Weight regain | 67 | <11 | N/A | <17 | 12.25 | 45.42 (14.14) | 45 [34, 57] | <11 (<16%) | 62 (92.5%) | 5 (7.5%) | <11 (<1%) | 0 (0.0%) |
| (Tirzepatide) | **Zepbound** | Moderate Responder | 1338 | 407 | 931 | 30.4 | 45.82 | 48.33 (13.02) | 48 [39, 57] | 21 (1.6%) | 1132 (84.6%) | 117 (8.7%) | 23 (1.7%) | 66 (5.0%) |
| (Tirzepatide) | **Zepbound** | Super Responder | 1118 | 196 | 922 | 17.5 | 103.81 | 46.04 (12.36) | 47 [37, 55] | 13 (1.2%) | 997 (89.2%) | 48 (4.3%) | 17 (1.5%) | 56 (5.1%) |

**Table S3.** Baseline metabolic conditions and longitudinal weight and BMI measurements before and after GLP-1RA therapy in non-propensity-matched cohorts of weight-loss super responders, moderate responders, the minimal weight-loss group, and the weight regain group across GLP-1RA drugs and brands.

| **DRUG (Active Compound)** | **BRAND (Marketed Formulation)** | **Weight-**  **Loss Outcome Category** | **Number of Patients (N)** | **With T2DM (N)** | **Without T2DM (N)** | **T2DM Percentage (%)** | **T2DM Association –log₁₀(P)** | **Baseline BMI in kg/m², Mean (SD)** | **Baseline BMI in kg/m², Median [IQR]** | **Baseline Weight in kg, Mean (SD)** | **Baseline Weight in kg, Median [IQR]** | **Pre-GLP1RA Weight Measurements, Mean (SD)** | **Pre-GLP1RA Weight Measurements, Median [IQR]** | **Post-GLP1AR Weight Measurements, Mean (SD)** | **Post-GLP1RA Weight Measurements, Median [IQR]** |
| --- | --- | --- | --- | --- | --- | --- | --- | --- | --- | --- | --- | --- | --- | --- | --- |
| **All GLP-1 Receptor Agonists** |  | Minimal weight-loss group | 63753 | 24647 | 39106 | 38.7 | inf | 35.99 (5.01) | 36.80 [32.20, 41.00] | 110.48 (26.25) | 106.98 [91.41, 126.25] | 28.78 (30.46) | 21 [8, 39] | 6.80 (5.85) | 5 [4, 9] |
| **All GLP-1 Receptor Agonists** |  | Weight regain | 7365 | 2355 | 5010 | 32 | 209.42 | 35.51 (5.08) | 36.05 [31.60, 41.00] | 106.06 (24.34) | 102.73 [87.93, 121.17] | 32.14 (34.96) | 23 [10, 42] | 10.86 (11.92) | 8 [5, 13] |
| **All GLP-1 Receptor Agonists** |  | Moderate Responder | 47196 | 17875 | 29321 | 37.9 | inf | 36.20 (4.73) | 36.90 [32.60, 41.00] | 108.24 (23.59) | 105.69 [90.96, 122.85] | 33.48 (34.58) | 25 [11, 45] | 8.13 (7.49) | 6 [4, 10] |
| **All GLP-1 Receptor Agonists** |  | Super Responder | 16950 | 3746 | 13204 | 22.1 | inf | 36.20 (4.31) | 36.50 [32.80, 41.00] | 105.76 (22.28) | 102.35 [89.31, 119.16] | 32.76 (32.99) | 24 [11, 44] | 8.79 (8.88) | 7 [4, 10] |
| **Semaglutide** | (Ozempic / Wegovy / Rybelsus) | Minimal weight-loss group | 16122 | 6527 | 9595 | 40.5 | 128.26 | 35.57 (5.17) | 36.10 [31.60, 41.00] | 107.71 (25.29) | 104.43 [89.51, 122.04] | 30.41 (30.18) | 22 [10, 40] | 6.58 (5.73) | 5 [4, 8] |
| **Semaglutide** | (Ozempic / Wegovy / Rybelsus) | Weight regain | 1960 | 664 | 1296 | 33.9 | 45.51 | 35.09 (5.09) | 35.30 [31.12, 41.00] | 104.61 (24.17) | 101.08 [86.95, 118.73] | 34.50 (36.82) | 24 [11, 45] | 10.63 (11.04) | 8 [5, 12] |
| **Semaglutide** | (Ozempic / Wegovy / Rybelsus) | Moderate Responder | 14819 | 6297 | 8522 | 42.5 | 73.9 | 35.75 (4.83) | 36.20 [32.00, 41.00] | 105.70 (22.96) | 103.25 [88.91, 119.50] | 36.01 (38.30) | 27 [13, 47] | 8.06 (7.33) | 6 [4, 9] |
| **Semaglutide** | (Ozempic / Wegovy / Rybelsus) | Super Responder | 4601 | 1212 | 3389 | 26.3 | 225.28 | 35.59 (4.37) | 35.60 [32.00, 41.00] | 103.44 (21.82) | 99.96 [87.53, 116.01] | 35.45 (35.22) | 26 [12, 47] | 9.33 (9.28) | 7 [5, 11] |
| **Tirzepatide** | (Mounjaro / Zepbound) | Minimal weight-loss group | 4474 | 1133 | 3341 | 25.3 | 238.24 | 36.17 (5.21) | 37.40 [32.38, 41.00] | 112.28 (27.26) | 108.69 [92.13, 129.18] | 28.99 (30.55) | 21 [9, 39] | 5.89 (4.36) | 5 [3, 7] |
| **Tirzepatide** | (Mounjaro / Zepbound) | Weight regain | 794 | 146 | 648 | 18.4 | 70.27 | 34.06 (4.76) | 34.05 [30.62, 37.85] | 100.69 (21.71) | 97.42 [85.67, 113.95] | 28.58 (25.98) | 23 [10, 38] | 8.46 (7.83) | 6 [4, 10] |
| **Tirzepatide** | (Mounjaro / Zepbound) | Moderate Responder | 7345 | 2165 | 5180 | 29.5 | 270.39 | 36.30 (4.55) | 36.80 [32.80, 41.00] | 109.05 (23.85) | 106.71 [91.24, 123.73] | 30.65 (29.40) | 23 [11, 41] | 6.50 (5.34) | 5 [4, 8] |
| **Tirzepatide** | (Mounjaro / Zepbound) | Super Responder | 5367 | 1036 | 4331 | 19.3 | inf | 36.10 (4.22) | 36.30 [32.90, 41.00] | 105.62 (22.22) | 102.18 [89.13, 119.27] | 30.68 (29.79) | 23 [11, 41] | 7.13 (6.36) | 6 [4, 8] |
| (Semaglutide) | **Ozempic** | Minimal weight-loss group | 8436 | 4390 | 4046 | 52 | 3.74 | 35.20 (5.11) | 35.80 [31.20, 41.00] | 108.39 (25.01) | 105.85 [90.91, 122.03] | 30.91 (31.28) | 22 [9, 41] | 6.65 (6.13) | 5 [4, 8] |
| (Semaglutide) | **Ozempic** | Weight regain | 1006 | 466 | 540 | 46.3 | 1.71 | 34.52 (5.56) | 34.80 [30.30, 41.00] | 104.25 (24.69) | 100.88 [86.03, 118.83] | 36.63 (39.93) | 25 [12, 46] | 11.58 (12.39) | 8 [5, 14] |
| (Semaglutide) | **Ozempic** | Moderate Responder | 8278 | 4747 | 3531 | 57.3 | 40.01 | 35.39 (4.93) | 35.95 [31.70, 41.00] | 105.62 (22.81) | 103.62 [89.08, 119.54] | 38.53 (42.15) | 29 [14, 50] | 8.37 (7.82) | 6 [4, 10] |
| (Semaglutide) | **Ozempic** | Super Responder | 1941 | 957 | 984 | 49.3 | 0.27 | 35.76 (4.53) | 36.15 [32.35, 41.00] | 104.66 (23.39) | 100.43 [87.64, 119.26] | 39.98 (39.22) | 28 [14, 54] | 10.68 (10.83) | 8 [5, 13] |
| (Tirzepatide) | **Mounjaro** | Minimal weight-loss group | 2410 | 962 | 1448 | 39.9 | 22.38 | 35.54 (5.50) | 36.55 [31.82, 41.00] | 111.31 (27.33) | 107.87 [91.56, 128.07] | 28.01 (31.40) | 20 [8, 38] | 5.91 (4.49) | 5 [3, 7] |
| (Tirzepatide) | **Mounjaro** | Weight regain | 452 | 120 | 332 | 26.5 | 22.69 | 34.57 (5.20) | 35.40 [31.38, 38.70] | 100.20 (21.81) | 97.83 [84.48, 113.87] | 28.12 (27.27) | 21 [10, 35] | 8.71 (8.88) | 6 [4, 10] |
| (Tirzepatide) | **Mounjaro** | Moderate Responder | 3565 | 1741 | 1824 | 48.8 | 0.78 | 35.90 (4.74) | 36.25 [32.40, 41.00] | 108.99 (23.64) | 106.77 [91.69, 123.64] | 32.04 (30.97) | 24 [11, 43] | 6.96 (6.42) | 5 [4, 8] |
| (Tirzepatide) | **Mounjaro** | Super Responder | 2039 | 812 | 1227 | 39.8 | 19.41 | 36.21 (4.57) | 37.15 [32.88, 41.00] | 106.69 (23.40) | 103.42 [88.58, 121.84] | 33.67 (34.26) | 24 [11, 45] | 8.08 (8.72) | 6 [4, 10] |
| (Semaglutide) | **Wegovy** | Minimal weight-loss group | 1468 | 45 | 1423 | 3.1 | 282.54 | 36.99 (4.54) | 38.20 [33.10, 41.00] | 110.16 (25.96) | 105.33 [91.41, 125.42] | 30.04 (28.96) | 23 [11, 41] | 5.78 (4.33) | 4 [3, 7] |
| (Semaglutide) | **Wegovy** | Weight regain | 239 | <11 | N/A | <5 | 47.26 | 35.45 (4.65) | 35.10 [31.88, 41.00] | 104.44 (23.04) | 101.01 [87.39, 116.07] | 31.94 (27.95) | 24 [11, 46] | 8.50 (8.30) | 6 [4, 9] |
| (Semaglutide) | **Wegovy** | Moderate Responder | 1936 | 49 | 1887 | 2.5 | inf | 36.38 (4.58) | 36.90 [32.50, 41.00] | 107.37 (22.78) | 104.63 [90.31, 120.82] | 30.35 (28.41) | 24 [11, 40] | 6.10 (4.76) | 5 [3, 7] |
| (Semaglutide) | **Wegovy** | Super Responder | 1258 | 23 | 1235 | 1.8 | 255.19 | 35.41 (4.25) | 35.20 [31.90, 39.30] | 101.10 (19.85) | 97.43 [86.83, 111.89] | 30.00 (26.36) | 23 [12, 40] | 6.84 (5.84) | 5 [4, 8] |
| (Tirzepatide) | **Zepbound** | Minimal weight-loss group | 754 | <11 | N/A | <2 | 156.68 | 36.94 (4.81) | 39.05 [32.22, 41.00] | 114.43 (27.35) | 110.39 [94.33, 130.89] | 32.87 (32.85) | 25 [10, 44] | 5.82 (4.01) | 4 [3, 7] |
| (Tirzepatide) | **Zepbound** | Weight regain | 67 | <11 | N/A | <17 | 13.04 | 33.99 (5.07) | 33.80 [30.52, 37.55] | 98.61 (22.04) | 92.19 [83.47, 106.95] | 29.52 (20.93) | 26 [12, 40] | 9.07 (4.95) | 8 [6, 11] |
| (Tirzepatide) | **Zepbound** | Moderate Responder | 1338 | 24 | 1314 | 1.8 | 271.72 | 35.78 (4.50) | 36.00 [32.35, 41.00] | 107.81 (23.38) | 104.76 [89.79, 122.71] | 28.71 (27.21) | 22 [10, 39] | 5.70 (3.53) | 5 [4, 7] |
| (Tirzepatide) | **Zepbound** | Super Responder | 1118 | <11 | N/A | <1 | 235.78 | 35.52 (4.03) | 35.20 [32.20, 40.03] | 103.78 (21.24) | 100.24 [88.85, 116.17] | 25.68 (23.08) | 19 [10, 35] | 6.28 (3.70) | 6 [4, 8] |

**Table S4.** Duration and number of prescriptions, number of clinical documents per patient per month among non-propensity-matched cohorts of weight loss super responders, moderate responders, minimal weight-loss group, and weight regain.

| **DRUG (Active Compound)** | **BRAND (Marketed Formulation)** | **Weight-Loss Outcome Category** | **Number of Patients (N)** | **Duration of prescriptions per patient in months, Mean (SD)** | **Duration of prescriptions per patient in months, Median [IQR]** | **Number of prescriptions per patient, Mean (SD)** | **Number of prescriptions per patient, Median [IQR]** | **Baseline monthly document per patient, Mean (SD)** | **Baseline monthly documents per patient, Median [IQR]** | **Post-treatment monthly documents per patient, Mean (SD)** | **Post-treatment monthly documents per patient, Median [IQR]** |
| --- | --- | --- | --- | --- | --- | --- | --- | --- | --- | --- | --- |
| **All GLP-1 Receptor Agonists** |  | Minimal weight-loss group | 63753 | 27.90 (26.84) | 21.28 [10.62, 36.59] | 13.24 (11.50) | 10 [6, 18] | 7.6 (16.9) | 3 [1, 7] | 6.2 (11.7) | 3 [2, 7] |
| **All GLP-1 Receptor Agonists** |  | Weight regain | 7365 | 25.19 (22.76) | 20.60 [11.60, 31.95] | 14.89 (13.00) | 11 [6, 20] | 8.3 (19.7) | 3 [2, 8] | 8.8 (22.8) | 4 [2, 8] |
| **All GLP-1 Receptor Agonists** |  | Moderate Responder | 47196 | 22.08 (20.30) | 17.65 [8.99, 28.72] | 15.02 (11.85) | 11 [7, 20] | 7.3 (16.7) | 3 [1, 7] | 6.2 (12.9) | 3 [2, 6] |
| **All GLP-1 Receptor Agonists** |  | Super Responder | 16950 | 18.23 (13.51) | 15.54 [9.16, 24.65] | 17.73 (13.04) | 15 [9, 23] | 6.7 (16.4) | 3 [1, 6] | 6.2 (15.9) | 3 [2, 6] |
| **Semaglutide** | (Ozempic / Wegovy / Rybelsus) | Minimal weight-loss group | 16122 | 17.16 (13.08) | 14.15 [6.71, 24.01] | 9.77 (7.47) | 8 [4, 13] | 7.2 (15.8) | 3 [1, 7] | 5.8 (10.9) | 3 [2, 6] |
| **Semaglutide** | (Ozempic / Wegovy / Rybelsus) | Weight regain | 1960 | 16.84 (12.37) | 14.56 [7.16, 23.14] | 10.91 (8.23) | 9 [6, 15] | 7.9 (18.5) | 3 [2, 7] | 8.4 (20.2) | 4 [2, 7] |
| **Semaglutide** | (Ozempic / Wegovy / Rybelsus) | Moderate Responder | 14819 | 16.76 (12.11) | 13.90 [7.29, 23.33] | 11.51 (8.48) | 9 [6, 15] | 6.9 (16.1) | 3 [2, 6] | 6.0 (12.0) | 3 [2, 6] |
| **Semaglutide** | (Ozempic / Wegovy / Rybelsus) | Super Responder | 4601 | 16.76 (10.93) | 14.74 [8.50, 22.91] | 13.54 (9.57) | 11 [6, 18] | 6.7 (18.5) | 3 [2, 6] | 6.5 (15.8) | 3 [2, 6] |
| **Tirzepatide** | (Mounjaro / Zepbound) | Minimal weight-loss group | 4474 | 11.10 (7.95) | 8.90 [4.70, 15.95] | 10.69 (8.50) | 8 [5, 14] | 7.2 (15.2) | 3 [1, 7] | 5.1 (7.7) | 3 [1, 6] |
| **Tirzepatide** | (Mounjaro / Zepbound) | Weight regain | 794 | 14.44 (8.90) | 13.27 [7.11, 21.46] | 14.85 (11.13) | 12 [6, 20] | 6.8 (12.9) | 3 [1, 7] | 5.6 (11.7) | 3 [1, 6] |
| **Tirzepatide** | (Mounjaro / Zepbound) | Moderate Responder | 7345 | 11.70 (7.73) | 9.76 [5.81, 16.57] | 14.29 (10.34) | 12 [7, 19] | 6.2 (13.5) | 3 [1, 6] | 4.9 (8.2) | 3 [1, 6] |
| **Tirzepatide** | (Mounjaro / Zepbound) | Super Responder | 5367 | 13.33 (7.80) | 11.57 [7.59, 18.42] | 17.58 (12.29) | 15 [10, 23] | 6.0 (13.3) | 3 [1, 6] | 4.7 (8.7) | 3 [1, 6] |
| (Semaglutide) | **Ozempic** | Minimal weight-loss group | 8436 | 16.98 (13.23) | 13.71 [6.72, 23.75] | 9.46 (7.04) | 8 [4, 11] | 7.7 (16.8) | 3 [2, 7] | 5.8 (11.3) | 3 [2, 7] |
| (Semaglutide) | **Ozempic** | Weight regain | 1006 | 17.16 (12.75) | 14.30 [7.83, 23.52] | 10.00 (7.90) | 8 [5, 13] | 8.8 (20.1) | 3 [2, 8] | 9.1 (20.7) | 4 [2, 8] |
| (Semaglutide) | **Ozempic** | Moderate Responder | 8278 | 17.03 (12.45) | 14.00 [7.36, 23.81] | 10.65 (7.62) | 9 [6, 14] | 7.7 (17.2) | 3 [2, 7] | 6.3 (12.0) | 3 [2, 6] |
| (Semaglutide) | **Ozempic** | Super Responder | 1941 | 17.20 (12.02) | 14.55 [8.07, 24.10] | 11.39 (8.04) | 9 [6, 15] | 8.2 (23.4) | 4 [2, 7] | 8.1 (19.4) | 4 [2, 7] |
| (Tirzepatide) | **Mounjaro** | Minimal weight-loss group | 2410 | 10.84 (7.85) | 8.49 [4.50, 15.66] | 10.25 (7.93) | 8 [5, 14] | 7.4 (16.0) | 3 [1, 7] | 5.1 (8.0) | 3 [1, 6] |
| (Tirzepatide) | **Mounjaro** | Weight regain | 452 | 12.34 (8.25) | 10.25 [6.25, 17.05] | 12.91 (9.17) | 11 [6, 17] | 7.1 (14.0) | 3 [1, 7] | 6.3 (14.5) | 3 [1, 6] |
| (Tirzepatide) | **Mounjaro** | Moderate Responder | 3565 | 12.11 (7.82) | 10.15 [5.95, 17.35] | 13.36 (9.60) | 11 [7, 17] | 6.9 (15.4) | 3 [1, 6] | 5.3 (9.3) | 3 [2, 6] |
| (Tirzepatide) | **Mounjaro** | Super Responder | 2039 | 14.27 (8.33) | 12.12 [7.77, 20.72] | 16.61 (11.81) | 14 [8, 22] | 7.2 (15.9) | 3 [1, 6] | 5.6 (10.2) | 3 [2, 6] |
| (Semaglutide) | **Wegovy** | Minimal weight-loss group | 1468 | 9.98 (7.81) | 7.56 [3.91, 14.30] | 8.04 (5.57) | 6 [5, 10] | 5.7 (12.5) | 3 [2, 6] | 4.5 (7.7) | 3 [1, 5] |
| (Semaglutide) | **Wegovy** | Weight regain | 239 | 10.81 (7.70) | 8.83 [5.17, 14.75] | 11.03 (8.21) | 9 [6, 13] | 4.6 (10.2) | 2 [1, 5] | 4.6 (9.2) | 2 [1, 5] |
| (Semaglutide) | **Wegovy** | Moderate Responder | 1936 | 11.04 (7.40) | 9.04 [5.44, 14.98] | 10.96 (7.05) | 9 [6, 14] | 4.0 (8.2) | 2 [1, 4] | 3.6 (7.8) | 2 [1, 3] |
| (Semaglutide) | **Wegovy** | Super Responder | 1258 | 13.62 (7.78) | 12.24 [7.96, 17.82] | 13.46 (8.33) | 11 [8, 17] | 3.9 (8.1) | 2 [1, 4] | 3.5 (7.3) | 2 [1, 3] |
| (Tirzepatide) | **Zepbound** | Minimal weight-loss group | 754 | 6.71 (4.12) | 5.82 [3.43, 9.53] | 8.51 (6.32) | 6 [4, 11] | 6.9 (14.8) | 3 [2, 7] | 5.4 (7.8) | 3 [2, 6] |
| (Tirzepatide) | **Zepbound** | Weight regain | 67 | 8.09 (4.76) | 8.22 [4.01, 11.73] | 12.21 (9.41) | 9 [6, 15] | 5.2 (8.4) | 3 [1, 5] | 4.3 (4.6) | 3 [2, 5] |
| (Tirzepatide) | **Zepbound** | Moderate Responder | 1338 | 7.22 (3.91) | 6.70 [4.19, 9.71] | 12.25 (8.42) | 10 [6, 16] | 5.5 (11.4) | 3 [1, 6] | 4.3 (5.7) | 3 [1, 5] |
| (Tirzepatide) | **Zepbound** | Super Responder | 1118 | 8.90 (3.75) | 8.94 [5.94, 11.70] | 15.27 (10.01) | 13 [8, 20] | 4.9 (8.8) | 3 [1, 5] | 4.1 (5.4) | 3 [1, 5] |

**Table S5. Demographic characteristics of age- and gender–propensity-matched cohorts of weight-loss super responders and the minimal weight-loss group across GLP-1RA drugs and brands.**

| **DRUG**  **(Active Compound)** | **BRAND**  **(Marketed Formulation)** | **Weight-Loss Outcome Category** | **Number of Patients (N)** | **Male (N)** | **Female (N)** | **Male Percentage (%)** | **Gender Association –log₁₀(P)** | **Age in years,**  **Mean ± SD (years)** | **Age,**  **Median [IQR] (years)** | **Pregnant Patients N (%)** | **Race: White N (%)** | **Race: Black/African American N (%)** | **Race: Hispanic N (%)** | **Race: Other/Unknown N (%)** |
| --- | --- | --- | --- | --- | --- | --- | --- | --- | --- | --- | --- | --- | --- | --- |
| **All GLP-1RA** |  | Minimal weight-loss group | 16948 | 3376 | 13572 | 19.9 | inf | 51.52 (13.57) | 52 [41, 62] | 209 (1.2%) | 14022 (82.7%) | 1474 (8.7%) | 541 (3.2%) | 1009 (5.9%) |
| **All GLP-1RA** |  | Super Responder | 16948 | 3375 | 13573 | 19.9 | inf | 51.52 (13.58) | 52 [41, 62] | 157 (0.9%) | 15230 (89.9%) | 749 (4.4%) | 282 (1.7%) | 737 (4.3%) |
| **Semaglutide** | (Ozempic / Wegovy / Rybelsus) | Minimal weight-loss group | 4599 | 889 | 3710 | 19.3 | inf | 53.48 (14.49) | 54 [43, 65] | 46 (1.0%) | 3905 (84.9%) | 344 (7.5%) | 162 (3.5%) | 242 (5.3%) |
| **Semaglutide** | (Ozempic / Wegovy / Rybelsus) | Super Responder | 4599 | 889 | 3710 | 19.3 | inf | 53.48 (14.48) | 54 [43, 65] | 41 (0.9%) | 4182 (90.9%) | 185 (4.0%) | 64 (1.4%) | 189 (4.1%) |
| **Tirzepatide** | (Mounjaro / Zepbound) | Minimal weight-loss group | 3857 | 1068 | 2789 | 27.7 | 168.29 | 51.99 (13.65) | 53 [42, 62] | 58 (1.5%) | 3096 (80.3%) | 388 (10.1%) | 113 (2.9%) | 259 (6.7%) |
| **Tirzepatide** | (Mounjaro / Zepbound) | Super Responder | 3857 | 1024 | 2833 | 26.5 | 185.8 | 51.66 (12.67) | 52 [43, 61] | 37 (1.0%) | 3420 (88.7%) | 188 (4.9%) | 72 (1.9%) | 178 (4.6%) |
| (Semaglutide) | **Ozempic** | Minimal weight-loss group | 1941 | 495 | 1446 | 25.5 | 102.61 | 59.11 (13.01) | 61 [50, 68] | 14 (0.7%) | 1633 (84.6%) | 137 (7.1%) | 68 (3.5%) | 92 (4.8%) |
| (Semaglutide) | **Ozempic** | Super Responder | 1941 | 495 | 1446 | 25.5 | 102.61 | 59.11 (13.01) | 61 [50, 68] | <11 (<1%) | 1748 (90.6%) | 84 (4.4%) | 27 (1.4%) | 71 (3.7%) |
| (Tirzepatide) | **Mounjaro** | Minimal weight-loss group | 1866 | 545 | 1321 | 29.2 | 71.43 | 54.59 (13.25) | 55 [45, 64] | 20 (1.1%) | 839 (85.5%) | 81 (8.3%) | N/A | 61 (6.2%) |
| (Tirzepatide) | **Mounjaro** | Super Responder | 1866 | 524 | 1342 | 28.1 | 79.24 | 53.93 (12.83) | 54 [45, 64] | 15 (0.8%) | 899 (91.6%) | 33 (3.4%) | N/A | 49 (5.0%) |
| (Semaglutide) | **Wegovy** | Minimal weight-loss group | 981 | 179 | 802 | 18.2 | 87.31 | 47.58 (14.20) | 48 [37, 57] | 15 (1.5%) | 1490 (79.8%) | 194 (10.4%) | 61 (3.3%) | 121 (6.5%) |
| (Semaglutide) | **Wegovy** | Super Responder | 981 | 179 | 802 | 18.2 | 87.31 | 47.26 (13.12) | 47 [38, 56] | 16 (1.6%) | 1658 (88.9%) | 99 (5.3%) | 34 (1.8%) | 75 (4.0%) |
| (Tirzepatide) | **Zepbound** | Minimal weight-loss group | 719 | 174 | 545 | 24.2 | 42.81 | 47.11 (13.44) | 47 [37, 57] | 19 (2.6%) | 585 (81.4%) | 66 (9.2%) | 15 (2.1%) | 53 (7.4%) |
| (Tirzepatide) | **Zepbound** | Super Responder | 719 | 154 | 565 | 21.4 | 52.3 | 47.03 (12.10) | 47 [38, 56] | <11 (<2%) | 634 (88.2%) | 27 (3.8%) | 14 (1.9%) | 44 (6.1%) |

**Table S6. Baseline metabolic conditions and longitudinal weight and BMI measurements before and after GLP-1RA therapy in age- and gender–propensity-matched cohorts of weight-loss super responders and minimal weight-loss group.**

| **DRUG**  **(Active Compound)** | **BRAND**  **(Marketed Formulation)** | **Weight-**  **Loss Outcome Category** | **Num. of Pts. (N)** | **With T2DM (N)** | **Without T2DM (N)** | **T2DM Percentage (%)** | **T2DM Association –log₁₀(P)** | **Baseline BMI in kg/m², Mean (SD)** | **Baseline BMI in kg/m², Median [IQR]** | **Baseline Weight in kg, Mean (SD)** | **Baseline Weight in kg, Median [IQR]** | **Pre-GLP1RA Weight Measurements, Mean (SD)** | **Pre-GLP1RA Weight Measurements, Median [IQR]** | **Post-GLP1AR Weight Measurements, Mean (SD)** | **Post-GLP1RA Weight Measurements, Median [IQR]** |
| --- | --- | --- | --- | --- | --- | --- | --- | --- | --- | --- | --- | --- | --- | --- | --- |
| **All GLP-1 Receptor Agonists** |  | Minimal weight-loss group | 16948 | 6950 | 9998 | 41 | 120.5 | 36.55 (4.94) | 37.90 [33.00, 41.00] | 108.49 (25.62) | 105.07 [89.88, 123.79] | 26.03 (28.91) | 18 [6, 36] | 7.05 (6.21) | 5 [4, 9] |
| **All GLP-1 Receptor Agonists** |  | Super Responder | 16948 | 3746 | 13202 | 22.1 | inf | 36.20 (4.31) | 36.50 [32.80, 41.00] | 105.75 (22.28) | 102.35 [89.31, 119.16] | 32.76 (32.99) | 24 [11, 44] | 8.79 (8.88) | 7 [4, 10] |
| **Semaglutide** | (Ozempic / Wegovy / Rybelsus) | Minimal weight-loss group | 4599 | 1849 | 2750 | 40.2 | 39.55 | 36.16 (5.20) | 37.20 [32.10, 41.00] | 105.16 (24.73) | 102.23 [87.27, 119.27] | 30.46 (30.32) | 22 [10, 42] | 6.60 (5.81) | 5 [4, 8] |
| **Semaglutide** | (Ozempic / Wegovy / Rybelsus) | Super Responder | 4599 | 1212 | 3387 | 26.4 | 224.97 | 35.59 (4.38) | 35.60 [32.00, 41.00] | 103.43 (21.82) | 99.93 [87.53, 116.01] | 35.45 (35.22) | 26 [12, 47] | 9.33 (9.29) | 7 [5, 11] |
| **Tirzepatide** | (Mounjaro / Zepbound) | Minimal weight-loss group | 3857 | 928 | 2929 | 24.1 | 227.03 | 36.17 (5.21) | 37.40 [32.38, 41.00] | 110.57 (26.72) | 106.87 [90.75, 127.13] | 28.74 (29.73) | 21 [9, 39] | 5.92 (4.43) | 5 [3, 7] |
| **Tirzepatide** | (Mounjaro / Zepbound) | Super Responder | 3857 | 855 | 3002 | 22.2 | 261.16 | 36.17 (4.20) | 36.30 [32.90, 41.00] | 106.77 (22.79) | 103.54 [89.53, 121.20] | 30.28 (29.52) | 23 [11, 40] | 7.44 (6.84) | 6 [4, 9] |
| (Semaglutide) | **Ozempic** | Minimal weight-loss group | 1941 | 983 | 958 | 50.6 | 0.24 | 35.72 (5.08) | 36.40 [31.40, 41.00] | 104.53 (24.40) | 102.35 [87.65, 117.73] | 32.16 (33.00) | 24 [11, 43] | 7.10 (6.32) | 6 [4, 9] |
| (Semaglutide) | **Ozempic** | Super Responder | 1941 | 957 | 984 | 49.3 | 0.27 | 35.76 (4.53) | 36.15 [32.35, 41.00] | 104.66 (23.39) | 100.43 [87.64, 119.26] | 39.98 (39.22) | 28 [14, 54] | 10.68 (10.83) | 8 [5, 13] |
| (Tirzepatide) | **Mounjaro** | Minimal weight-loss group | 1866 | 712 | 1154 | 38.2 | 23.85 | 35.65 (5.56) | 36.70 [32.00, 41.00] | 108.25 (25.92) | 104.10 [90.02, 123.82] | 27.52 (30.18) | 19 [8, 37] | 5.95 (4.62) | 5 [3, 7] |
| (Tirzepatide) | **Mounjaro** | Super Responder | 1866 | 745 | 1121 | 39.9 | 17.5 | 36.28 (4.58) | 37.40 [32.90, 41.00] | 107.26 (23.45) | 104.27 [89.04, 122.53] | 33.47 (34.11) | 24 [11, 45] | 8.23 (9.01) | 6 [4, 10] |
| (Semaglutide) | **Wegovy** | Minimal weight-loss group | 981 | 35 | 946 | 3.6 | 185.27 | 36.98 (4.61) | 38.20 [32.90, 41.00] | 109.51 (25.64) | 105.27 [89.88, 124.61] | 31.82 (30.72) | 24 [11, 43] | 5.71 (4.35) | 4 [3, 7] |
| (Semaglutide) | **Wegovy** | Super Responder | 981 | 19 | 962 | 1.9 | 198.42 | 35.60 (4.22) | 35.40 [32.10, 39.70] | 102.48 (20.33) | 99.05 [88.03, 113.29] | 28.89 (26.21) | 23 [11, 38] | 7.03 (6.34) | 5 [4, 8] |
| (Tirzepatide) | **Zepbound** | Minimal weight-loss group | 719 | <11 | 710 | <2 | 149.93 | 36.94 (4.81) | 39.05 [32.22, 41.00] | 114.37 (27.66) | 110.36 [93.92, 131.23] | 32.24 (31.76) | 24 [10, 44] | 5.80 (4.02) | 4 [3, 7] |
| (Tirzepatide) | **Zepbound** | Super Responder | 719 | <11 | 715 | <2 | 154.2 | 35.80 (3.88) | 35.50 [32.80, 41.00] | 105.08 (22.13) | 102.23 [89.43, 119.34] | 25.41 (21.29) | 20 [10, 36] | 6.55 (3.74) | 6 [4, 8] |

**Table S7. Duration and number of prescriptions, number of clinical documents per patient per month among age- and gender–propensity-matched cohorts of weight-loss super responders and minimal weight-loss group.**

| **DRUG**  **(Active Compound)** | **BRAND**  **(Marketed Formulation)** | **Weight-Loss Outcome Category** | **Number of Patients (N)** | **Duration of prescriptions per patient in months, Mean (SD)** | **Duration of prescriptions per patient in months, Median [IQR]** | **Number of prescriptions per patient, Mean (SD)** | **Number of prescriptions per patient, Median [IQR]** | **Baseline monthly document per patient, Mean (SD)** | **Baseline monthly documents per patient, Median [IQR]** | **Post-treatment monthly documents per patient, Mean (SD)** | **Post-treatment monthly documents per patient, Median [IQR]** |
| --- | --- | --- | --- | --- | --- | --- | --- | --- | --- | --- | --- |
| **All** | **All** | Minimal weight-loss group | 16948 | 49.40 (37.05) | 43.64 [25.60, 65.86] | 16.88 (15.04) | 13 [6, 24] | 7.8 (16.9) | 3 [1, 8] | 7.4 (15.1) | 3 [2, 7] |
| **All** | **All** | Super Responder | 16948 | 18.23 (13.51) | 15.54 [9.16, 24.65] | 17.73 (13.04) | 15 [9, 23] | 6.7 (16.4) | 3 [1, 6] | 6.2 (15.9) | 3 [2, 6] |
| **Semaglutide** | (Ozempic, Wegovy, Rybelsus) | Minimal weight-loss group | 4599 | 24.86 (16.29) | 23.68 [11.11, 36.08] | 11.03 (8.83) | 9 [5, 15] | 6.6 (15.1) | 3 [1, 6] | 6.2 (13.5) | 3 [2, 6] |
| **Semaglutide** | (Ozempic, Wegovy, Rybelsus) | Super Responder | 4599 | 16.76 (10.94) | 14.73 [8.50, 22.91] | 13.54 (9.58) | 11 [6, 18] | 7.2 (19.4) | 3 [2, 6] | 6.9 (16.5) | 3 [2, 6] |
| **Tirzepatide** | (Mounjaro, Zepbound) | Minimal weight-loss group | 3857 | 11.75 (8.20) | 9.96 [4.89, 17.28] | 10.87 (8.80) | 8 [5, 14] | 7.1 (14.9) | 3 [1, 7] | 5.0 (7.2) | 3 [1, 6] |
| **Tirzepatide** | (Mounjaro, Zepbound) | Super Responder | 3857 | 15.64 (7.93) | 14.11 [9.72, 21.66] | 19.66 (13.26) | 17 [10, 26] | 6.9 (14.6) | 3 [1, 7] | 5.1 (9.1) | 3 [1, 5] |
| (Semaglutide) | **Ozempic** | Minimal weight-loss group | 1941 | 27.02 (16.92) | 26.46 [12.67, 39.21] | 11.41 (9.09) | 9 [4, 15] | 7.6 (17.8) | 3 [1, 7] | 6.7 (16.1) | 3 [2, 6] |
| (Semaglutide) | **Ozempic** | Super Responder | 1941 | 17.20 (12.02) | 14.55 [8.07, 24.10] | 11.39 (8.04) | 9 [6, 15] | 8.2 (23.4) | 4 [2, 7] | 8.1 (19.4) | 4 [2, 7] |
| (Tirzepatide) | **Mounjaro** | Minimal weight-loss group | 1866 | 11.53 (8.28) | 9.17 [4.55, 17.45] | 10.27 (8.16) | 8 [4, 13] | 7.3 (15.9) | 3 [1, 7] | 4.9 (7.5) | 3 [1, 6] |
| (Tirzepatide) | **Mounjaro** | Super Responder | 1866 | 14.96 (8.35) | 13.03 [8.42, 21.75] | 17.05 (12.08) | 15 [9, 23] | 7.7 (16.3) | 3 [1, 7] | 5.5 (9.2) | 3 [2, 6] |
| (Semaglutide) | **Wegovy** | Minimal weight-loss group | 981 | 10.89 (8.36) | 8.31 [4.12, 15.80] | 8.71 (5.78) | 8 [5, 11] | 5.1 (12.7) | 2 [1, 5] | 4.0 (7.5) | 2 [1, 4] |
| (Semaglutide) | **Wegovy** | Super Responder | 981 | 15.24 (7.98) | 14.19 [9.53, 20.16] | 14.11 (8.93) | 12 [8, 18] | 4.9 (10.3) | 2 [1, 5] | 3.7 (6.4) | 2 [1, 4] |
| (Tirzepatide) | **Zepbound** | Minimal weight-loss group | 719 | 6.72 (4.13) | 5.85 [3.44, 9.58] | 8.59 (6.42) | 6 [4, 11] | 6.8 (13.9) | 3 [2, 7] | 5.3 (7.6) | 3 [2, 6] |
| (Tirzepatide) | **Zepbound** | Super Responder | 719 | 9.99 (3.72) | 10.50 [7.60, 12.71] | 17.28 (10.84) | 16 [10, 24] | 5.4 (9.3) | 3 [1, 6] | 4.3 (5.5) | 3 [1, 5] |


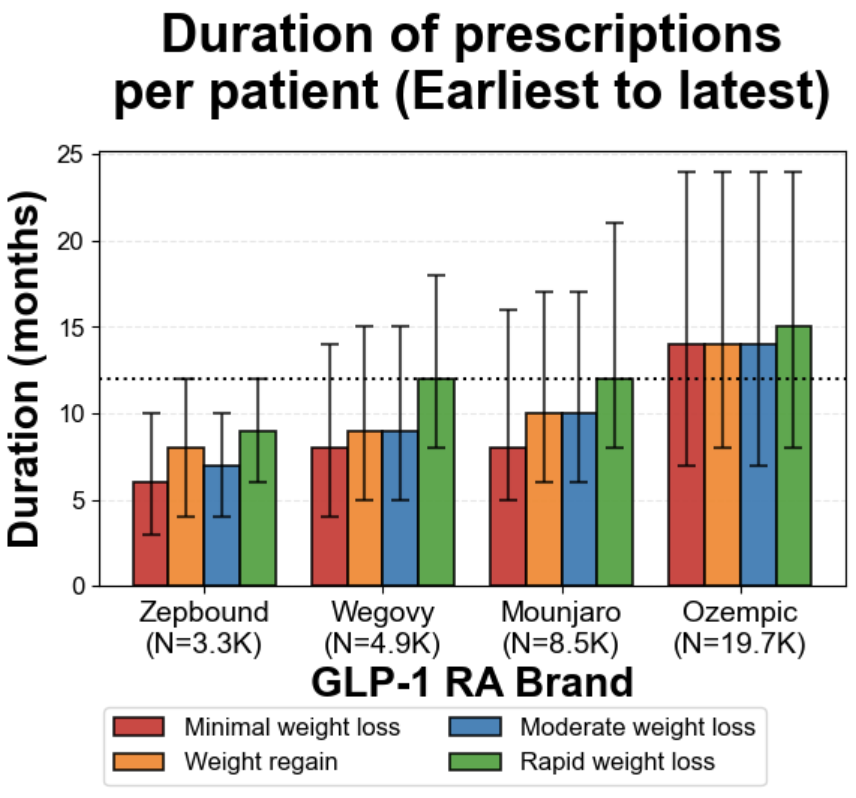

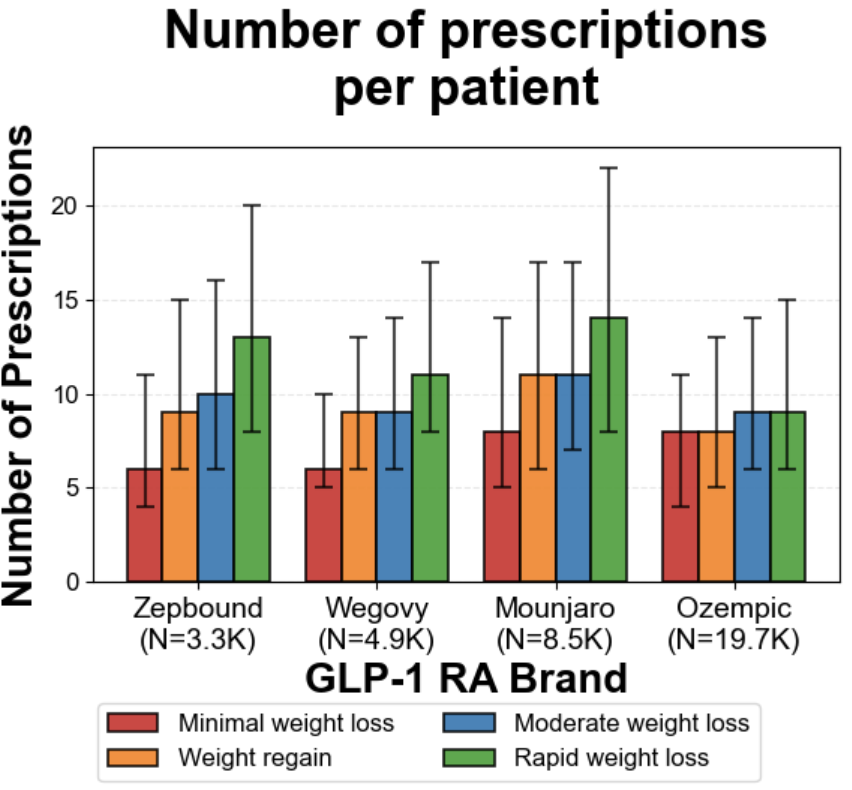


**Figure S1. The four categories examined are the minimal weight loss group (red), weight regain group (orange), moderate responders (moderate weight loss; blue) and super responders (rapid weight loss). (Left)** Median duration of GLP-1RA brand-level prescriptions per patient for each response category (first prescription - index date, to last prescription even if beyond the observation period of 1 year post first prescription). Dotted line indicates 12-month threshold. **(Right)** Median number of GLP-1RA brand-level prescriptions per patient for each response category. In both plots, error bars represent interquartile range (IQR).
